# Association between serum phosphate levels within the normal range and all-cause mortality among US adults

**DOI:** 10.1210/jendso/bvag021

**Published:** 2026-01-28

**Authors:** Mayuko Yamamoto, Sho Katsuragawa, Yuichi Takashi, Dai Nagata, Kyoko Toyokawa, Kosuke Inoue, Daiji Kawanami

**Affiliations:** Department of Endocrinology and Diabetes, Fukuoka University School of Medicine, Fukuoka 814-0180, Japan; Centre for Endocrinology and Reproductive Health, Hudson Institute of Medical Research, Clayton, VIC 3168, Australia; Department of Endocrinology and Metabolism, Graduate School of Medicine, Yokohama City University, Yokohama 236-0004, Japan; Department of Endocrinology and Diabetes, Fukuoka University School of Medicine, Fukuoka 814-0180, Japan; Department of Endocrinology and Diabetes, Fukuoka University School of Medicine, Fukuoka 814-0180, Japan; Department of Endocrinology and Diabetes, Fukuoka University School of Medicine, Fukuoka 814-0180, Japan; Department of Health Promotion and Behavioral Sciences, Graduate School of Medicine and School of Public Health, Kyoto University, Kyoto 606-8501, Japan; Department of Endocrinology and Diabetes, Fukuoka University School of Medicine, Fukuoka 814-0180, Japan

**Keywords:** serum phosphate levels, chronic kidney disease, mortality, phosphate metabolism, hyperphosphatemia

## Abstract

**Introduction:**

Hyperphosphatemia is associated with increased mortality in chronic kidney disease (CKD), but the clinical relevance of variation within the normal serum phosphate range remains unclear. This study evaluated the association between higher-normal serum phosphate levels within the normal range and all-cause mortality among US adults.

**Methods:**

We conducted a cohort study using data from 15 848 individuals aged ≥20 years with serum phosphate levels between 2.5 and 4.5 mg/dL from the Third National Health and Nutrition Examination Survey (1988-1994) linked to mortality data ascertained by the National Center for Health Statistics with the National Death Index through December 2019. Baseline serum phosphate was the exposure. Participants were categorized into lower-normal (2.5 mg/dL to <3.5 mg/dL; n = 7691) and higher-normal (3.5 mg/dL to ≤4.5 mg/dL; n = 8157) serum phosphate groups. Cox proportional hazard models were used to estimate adjusted hazard ratios (aHR) for all-cause mortality. A subgroup analysis was conducted by CKD status (estimated glomerular filtration rate ≥60 and <60 mL/min/1.73 m^2^).

**Results:**

Over a median follow-up of 26.3 years, 6660 (42%) participants died. Compared to the lower-normal group, the higher-normal group showed no significant increase in all-cause mortality risk in the overall population (aHR 1.06 [95% CI, 0.98-1.13]). However, among individual with CKD, higher-normal serum phosphate was significantly associated with increased all-cause mortality (aHR 1.13 [95% CI, 1.02-1.25]), wheres no association was observed in those with estimated glomerular filtration rate ≥60 mL/min/1.73 m^2^ (aHR 1.01 [95% CI, 0.92-1.12]; *P* for interaction, .14).

**Conclusion:**

Higher-normal phosphate levels were associated with an increased risk of all-cause mortality among people with CKD.

Phosphate is an essential nutrient for the human body, with approximately 85% stored in the skeleton in the form of hydroxyapatite [1]. The remaining phosphate is distributed intracellularly (14%) and within the extracellular fluid (<1%). Phosphate plays diverse physiological roles. Intracellular phosphate is vital for biological processes, such as signal transduction, membrane synthesis, pH regulation, and energy metabolism, particularly through the synthesis of adenosine triphosphate [2]. Because extracellular phosphate is crucial for bone matrix mineralization, hypophosphatemia causes rickets/osteomalacia characterized by aberrant bone mineralization, whereas hyperphosphatemia induces ectopic calcification or ossification [3]. Therefore, serum phosphate levels are tightly regulated within a narrow range, typically between 2.5 and 4.5 mg/dL in adults [2].

Previous studies have consistently demonstrated that hyperphosphatemia is associated with increased risks of carotid atherosclerosis, coronary calcification, cardiovascular disease (CVD), and mortality, particularly in patients with chronic kidney disease (CKD) [4-11]. In addition, several studies have reported that higher serum phosphate levels are associated with an increased risk of CVD [12-14] and end-stage renal disease [15] in individuals without CKD. Accordingly, current nephrology guidelines recommend targets and treatment strategies aimed at correcting serum phosphate levels toward the normal range [16]. Although the systematic review included some analyses of serum phosphate levels within the normal range, most of the evidence was derived from examining phosphate levels outside the normal range—namely hypophosphatemia and hyperphosphatemia—and their associations with cardiovascular mortality and coronary artery disease [11]. Despite these insights, it remains unclear whether higher serum phosphate levels within the normal range are associated with long-term mortality risks.

To address this knowledge gap, using the nationally representative data in the United States with a 30-year follow-up, we investigated the association between higher-normal serum phosphate levels within the normal range and all-cause mortality. Given the previous evidence, we also assessed the relationship among adults with and without CKD.

## Materials and methods

### Data source and study population

**Figure 1:**
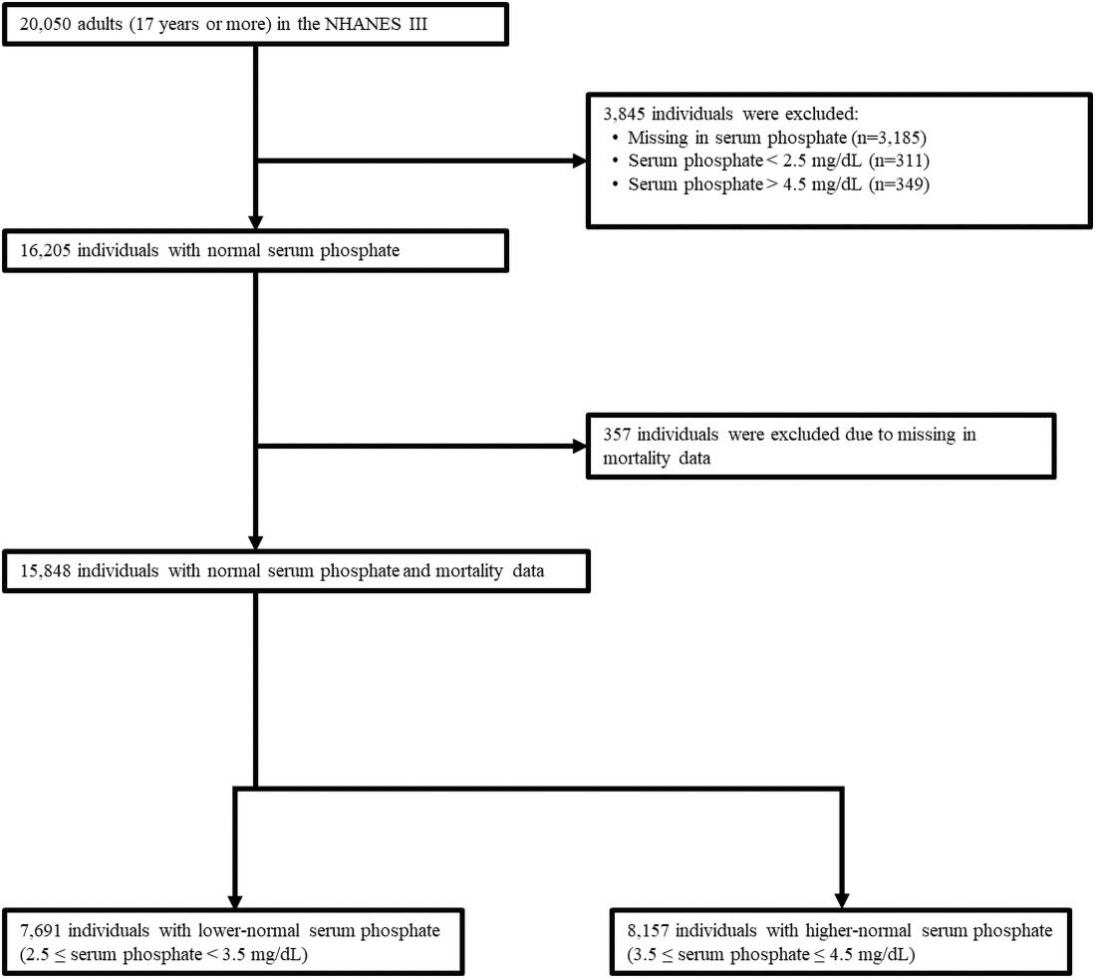
Flow of sample selection. A total of 20 050 adults participated in the Third National Health and Nutrition Examination Survey (NHANES III) from 1988 to 1994. Of these, 16 205 individuals with serum phosphate levels within the normal range (2.5-4.5 mg/dL) were included. After excluding 357 individuals with missing mortality data, 15 848 individuals were included in the final analysis. Mortality data were linked through December 2019.

We conducted a population-based cohort study using the Third National Health and Nutrition Examination Survey (NHANES III) and its linked national mortality data through December 2019. NHANES III was a nationwide survey to assess the health and nutritional status of the US population conducted from 1988 to 1994 [17]. It employed a stratified, multistage probability sampling design [18]. Data of structured interviews, physical examinations, and laboratory tests were available in NHANES III. From the data of 20 050 adults in NHANES III, we included 16 205 individuals whose serum phosphate levels were within the normal range (2.5-4.5 mg/dL) [19]. After excluding 357 individuals with missing mortality data, a total of 15 848 individuals were analyzed (Fig. 1).

### Exposure variable: serum phosphate level within a normal range

Our primary exposure was serum phosphate levels at the survey. In NHANES III, serum phosphate levels were measured with a Hitachi Model 737 multichannel analyzer (Boehringer Mannheim Diagnostics, Indianapolis, USA) [20]. We categorized 15 848 participants into 2 groups according to their serum phosphate levels using a median value of 3.5 mg/dL as the cutoff: lower-normal serum phosphate group (serum phosphate level: 2.5-<3.5 mg/dL; n = 7691) and higher-normal serum phosphate group (serum phosphate level: 3.5-≤4.5 mg/dL; n = 8157) (Fig. 1).

### Outcome variables

Our primary outcome was all-cause mortality, and the secondary outcome was cardiovascular mortality. These outcomes were ascertained by the National Center for Health Statistics with the National Death Index, with record matching by social security number, name, date of birth, race-ethnicity, sex, and state of residence. Cardiovascular mortality was defined as death from heart diseases and stroke using the International Classification of Diseases, 10th revision: acute rheumatic fever and chronic rheumatic heart diseases (I00-I09), hypertensive heart disease (I11), hypertensive heart and renal disease (I13), ischemic heart diseases (I20-I25), other heart diseases (I26-I51), and cerebrovascular diseases (I60-I69). Time to events was calculated from the date of serum phosphate measurement to the death date or the end of follow-up (ie, December 2019), whichever occurred earlier.

### Covariates

Sociodemographic characteristics including age (years), sex (male, female), race/ethnicity (non-Hispanic White, non-Hispanic Black, Mexican American, others), educational status (lower than high school, high school, higher than high school), and smoking status (never, former, current) were self-reported. Histories of diabetes, CVD (heart failure, heart attack, stroke), and cancer were also ascertained based on self-report. Anthropometric data and laboratory test data were measured based on the NHANES III manuals [20]. Body mass index (BMI) was calculated as weight in kilograms divided by height in meters squared. Estimated glomerular filtration rate (eGFR) was calculated using the Chronic Kidney Disease Epidemiology Collaboration equation: eGFR = 141 × min [serum creatinine/*k*, 1] × max [serum creatinine/*k*, 1] − 1.209 × 0.993 age × 1.018 [if female] × 1.159 [if Black]; *k* = 0.9 for male and 0.7 for female, *a* = −0.411 for male and −0.329 for female, and min indicates the minimum of serum creatinine/*k* or 1 and max indicates the maximum of serum creatinine/*k* or 1 [21]. Serum calcium level was adjusted when serum albumin level was less than 4.0 g/dL using a formula as follows: adjusted serum calcium (mg/dL) = calcium (mg/dL) – albumin (g/dL) + 4.0 [22].

### Statistical analyses

Continuous variables were presented as mean or median and categorical variables were presented as proportion according to serum phosphate levels. We applied multivariable Cox proportional hazard models adjusting for potential confounders to estimate adjusted hazard ratios (aHR) for all-cause and cardiovascular mortality for serum phosphate levels (higher-normal serum phosphate group vs lower-normal serum phosphate group [reference]). In model 1, age (continuous), age-squared (continuous), sex, race/ethnicity, educational status, smoking status, history of diabetes, history of cardiovascular disease, and history of cancer were included. Model 2 further included BMI (continuous), BMI-squared (continuous), eGFR (≥60, 30-60, <30 mL/min/1.73 m^2^), and adjusted serum calcium (continuous) in addition to the covariates in model 1. In each of model 1 and model 2, we excluded participants with missing data in the covariates: educational status (n = 106), smoking status (n = 1), history of diabetes (n = 17), history of cardiovascular disease (n = 204), history of cancer (n = 3), and BMI (n = 35). We then fitted restricted cubic spline models for Cox proportional hazard models with 3 knots at the 10th, 50th, and 90th percentiles of serum phosphate levels to investigate the association of continuous serum phosphate levels with all-cause mortality. Last, to assess the association of serum phosphate levels with mortality by CKD status, we incorporated an interaction term of serum phosphate levels and eGFR (≥60, <60 mL/min/1.73 m^2^) in our Cox proportional hazard models (model 2).

In all analyses, we applied the NHANES sampling weights to account for the differential probability of selecting the participants and the nonresponse of those eligible and approached [23]. All statistical analyses were performed using R software version 4.3.2. and Stata software version 15.

## Results

### Participants’ characteristics

Of a total of 15 848 participants, 7386 (46.6%) were male, and the mean age was 47.4 years (Table 1). Mean age was 49.6 years in the lower-normal group and 45.3 years in the higher-normal group. In the lower-normal serum phosphate group, 47.2% were female and 44.3% were of non-Hispanic White ethnicity, whereas the corresponding proportion in the higher-normal group were 59.2% and 39.4%, respectively. Histories of diabetes (8.1% vs 7.6%), hypertension (8.9% vs 24.7%), dyslipidemia (33.0% vs 34.6%), cardiovascular disease (9.0% vs 7.7%), and cancer (8.0% vs 6.8%) were also reported (Table 2). The mean BMI was 27.2 kg/m^2^ in the lower-normal group and 26.7 kg/m^2^ in the higher-normal group, and mean eGFR was 76.1 mL/min/1.73m^2^ and 79.0 mL/min/1.73m^2^, respectively.

**Table 1:** Sociodemographic characteristics according to serum phosphate levels, NHANES III

	Total	Serum phosphate levels (mg/dL)	
		Lower-normal (≤2.5, <3.5)	Higher-normal (≤3.5, ≤4.5)
	n = 15 848	n = 7691	n = 8157
Serum phosphate, mg/dL	3.5 (0.4)	3.1 (0.2)	3.8 (0.3)
Age, years	47.4 (20.0)	49.6 (19.8)	45.3 (20.1)
17-20	922 (5.8%)	265 (3.4%)	657 (8.1%)
21-40	6164 (38.9%)	2830 (36.8%)	3334 (40.9%)
41-60	3949 (24.9%)	1997 (26.0%)	1952 (23.9%)
61-80	3747 (23.6%)	2012 (26.2%)	1735 (21.3%)
≥81	1066 (6.7%)	587 (7.6%)	479 (5.9%)
Sex			
Male	7386 (46.6%)	4059 (52.8%)	3327 (40.8%)
Female	8462 (53.4%)	3632 (47.2%)	4830 (59.2%)
Ethnicity			
Non-Hispanic White	6623 (41.8%)	3407 (44.3%)	3216 (39.4%)
Non-Hispanic Black	4237 (26.7%)	2001 (26.0%)	2236 (27.4%)
Mexican American	4361 (27.5%)	1992 (25.9%)	2369 (29.0%)
Others	627 (4.0%)	291 (3.8%)	336 (4.1%).
Education			
Less than high school	3740 (23.8%)	1877 (24.6%)	1863 (23.0%)
High school	7604 (48.3%)	3613 (47.3%)	3991 (49.2%)
Higher than high school	4398 (27.9%)	2144 (28.1%)	2254 (27.8%)
Missing	106 (0.7%)	57 (0.7%)	49 (0.6%)
Smoking			
Never	8026 (50.6%)	3772 (49.0%)	4254 (52.2%)
Former	3822 (24.1%)	2075 (27.0%)	1747 (21.4%).
Current	3999 (25.2%)	1844 (24.0%)	2155 (26.4%).
Missing	1 (0.0%)	0 (0.0%)	1 (0.0%)

Data are presented as mean (SD), n (%), or median (interquartile range).

Abbreviation: NHANES III, US Third National Health and Nutrition Examination Survey.

**Table 2:** Clinical and biological characteristics according to serum phosphate levels, NHANES III

	Total	Serum phosphate levels (mg/dL)	
		Lower-normal (≤2.5, <3.5)	Higher-normal (≤3.5, ≤4.5)
	n = 15 848	n = 7691	n = 8157
Diabetes mellitus	1246 (7.9%)	623 (8.1%)	623 (7.6%)
Missing	18 (0.1%)	9 (0.1%)	9 (0.1%)
Hypertension	4204 (26.8%)	2206 (28.9%)	1998 (24.7%)
Missing	145 (0.9%)	65 (0.8%)	80 (1.0%)
Dyslipidemia	2691 (33.8%)	1309 (33.0%)	1382 (34.6%)
Missing	7884 (49.7%)	3721 (48.4%)	4163 (51.0%)
Cardiovascular disease	1302 (8.3%)	685 (9.0%)	617 (7.7%)
Missing	209 (1.3%)	94 (1.2%)	115 (1.4%)
Cancer	1175 (7.4%)	619 (8.0%)	556 (6.8%)
Missing	3 (0.0%)	1 (0.0%)	2 (0.0%)
Body mass index, kg/m^2^	27.0 (5.8)	27.2 (5.7)	26.7 (5.8)
<18.5	364 (2.3%)	147 (1.9%)	217 (2.7%)
≤18.5, < 25	6077 (38.4%)	2775 (36.2%)	3302 (40.6%)
≤25, < 30	5453 (34.5%)	2788 (36.3%)	2665 (32.8%)
≤30	3916 (24.8%)	1966 (25.6%)	1950 (24.0%)
Missing	38 (0.2%)	15 (0.2%)	23 (0.3%)
Systolic blood pressure, mm Hg	125.3 (19.8)	127.0 (19.8)	123.6 (19.8)
Missing	336 (2.1%)	161 (2.1%)	175 (2.1%)
Diastolic blood pressure, mm Hg	73.9 (10.7)	74.9 (10.7)	73.0 (10.6)
Missing	344 (2.2%)	162 (2.1%)	182 (2.2%)
Estimated glomerular filtration rate, mL/min/1.73 m^2^	77.6 (20.9)	76.1 (20.0)	79.0 (21.5)
≤60	12 626 (79.7%)	6049 (78.7%)	6577 (80.6%)
≤30, < 60	3081 (19.4%)	1588 (20.6%)	1493 (18.3%)
<30	141 (0.9%)	54 (0.7%)	87 (1.1%)
Adjusted serum calcium, mg/dL	9.4 (0.4)	9.3 (0.4)	9.4 (0.4)
Follow-up duration, years	26.3 [15.5-28.5]	26.1 [13.7-28.3]	26.5 [17.5-28.7]

Data are presented as mean (SD), n (%), or median (IQR).

Abbreviation: NHANES III, US Third National Health and Nutrition Examination Survey.

### Serum phosphate levels within the normal range and mortality

**Figure 2:**
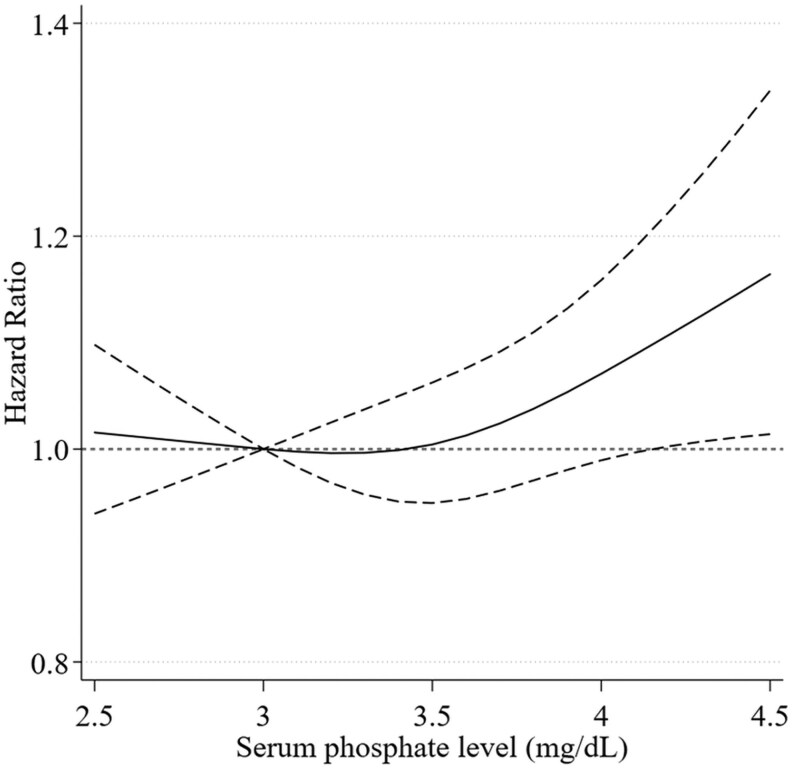
Association of serum phosphate level and all-cause mortality in the overall population using a restricted cubic spline model. The model adjusted for age, age-squared, sex, race/ethnicity, educational status, smoking status, history of diabetes, history of cardiovascular diseases, history of cancer, body mass index, body mass index-squared, estimated glomerular filtration rate, and adjusted serum calcium. The restricted cubic spline was modeled with 3 knots at the 10th, 50th, and 90th percentiles of serum phosphate level. The dash lines represent the 95% CIs for the spline model (reference is 3.0 mg/dL).

Over the median follow-up duration of 26.3 years, a total of 6660 (42.0%) all-cause mortality and 2507 (15.8%) cardiovascular mortality were ascertained. After adjusting for age and sex, the higher serum phosphate level within the normal range was associated with all-cause mortality (aHR 1.10 [95% CI, 1.02-1.18], Table 3). However, the association disappeared after adjusting for the potential confounders in model 1 (aHR 1.08 [95% CI, 1.00-1.15]) and model 2 (aHR 1.06 [95% CI, 0.98-1.13], Table 3). The serum phosphate levels within the normal range were not associated with cardiovascular mortality (Table 3). The restricted cubic spline of the overall population showed a trend of increased mortality with higher serum phosphate levels within the normal range, particularly for levels above 3.5 mg/dL (Fig. 2).

**Table 3:** Association of the serum phosphate levels with all-cause and cardiovascular mortality, NHANES III

	Serum phosphate level (mg/dL)	
	Lower-normal (≤2.5, <3.5)	Higher-normal (≤3.5, ≤4.5)
**All-cause mortality**		
**Unadjusted**		
Number analyzed	7691	8157
Number of events	3493	3167
Hazard ratio (95% CI)	Reference	0.91 (0.85-0.97)
**Age- and sex-adjusted**		
Number analyzed	7691	8157
Number of events	3493	3167
Hazard ratio (95% CI)	Reference	1.10 (1.02-1.18)
**Model 1** *^a^*		
Number analyzed	7634	8107
Number of events	3464	3143
Hazard ratio (95% CI)	Reference	1.08 (1.00-1.15)
**Model 2** *^b^*		
Number analyzed	7519	7963
Number of events	3411	3094
Hazard ratio (95% CI)	Reference	1.06 (0.98-1.13)
**Cardiovascular mortality**		
**Unadjusted**		
Number analyzed	7691	8157
Number of events	1318	1189
Hazard ratio (95% CI)	Reference	0.91 (0.81-1.03)
**Age- and sex-adjusted**		
Number analyzed	7691	8157
Number of events	1318	1189
Hazard ratio (95% CI)	Reference	1.11 (0.98-1.27)
**Model 1*^a^***		
Number analyzed	7634	8107
Number of events	1307	1180
Hazard ratio (95% CI)	Reference	1.10 (0.97-1.25)
**Model 2*^b^***		
Number analyzed	7519	7963
Number of events	1289	1163
Hazard ratio (95% CI)	Reference	1.08 (0.95-1.22)

Abbreviation: NHANES III, US Third National Health and Nutrition Examination Survey.

^*a*^Model 1 adjusted for age, age-squared, sex, race/ethnicity, educational status, and smoking status.

^*b*^Model 2 adjusted for age, age-squared, sex, race/ethnicity, educational status, smoking status, history of diabetes, history of cardiovascular diseases, history of cancer, body mass index, body mass index-squared, estimated glomerular filtration rate, and adjusted serum calcium.

### Subgroup analysis by CKD

**Figure 3:**
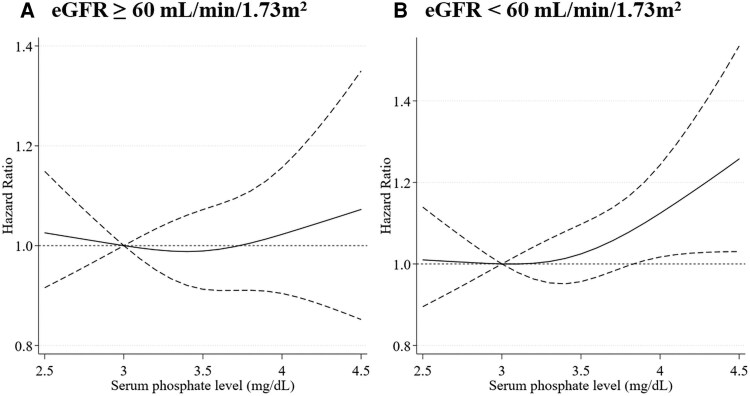
Association of serum phosphate level and all-cause mortality in subgroups stratified by participants’ renal function using a restricted cubic spline model. (A) Participants with eGFR ≥60 mL/min/1.73 m^2^. (B) Participants with eGFR <60 mL/min/1.73 m^2^. The model adjusted for age, age-squared, sex, race/ethnicity, educational status, smoking status, history of diabetes, history of cardiovascular diseases, history of cancer, body mass index, body mass index-squared, estimated glomerular filtration rate, and adjusted serum calcium. The restricted cubic spline was modeled with 3 knots at the 10th, 50th, and 90th percentiles of serum phosphate level. The dash lines represent the 95% CIs for the spline model (reference is 3.0 mg/dL).

We also conducted a subgroup analysis stratifying participants by eGFR. Clinical characteristics according to eGFR level (eGFR <30 mL/min/1.73 m^2^, 30 ≤ eGFR <60 mL/min/1.73 m^2^, eGFR ≥60 mL/min/1.73 m^2^) were presented in Table 4. Individuals with eGFR of <30 mL/min/1.73 m^2^ had a higher proportion of having a high phosphate level (4.1-4.5 mg/dL), history of diabetes, and history of CVD than those with eGFR of ≥60 mL/min/1.73 m^2^ or eGFR of ≥30 mL/min/1.73 m^2^ and <60 mL/min/1.73 m^2^. A total of 3732 (30.3%) deaths from all causes was observed among 12 327 participants with an eGFR of 60 mL/min/1.73 m^2^ or more, and 2773 (87.9%) deaths from all causes were observed among 3155 participants with eGFR < 60 mL/min/1.73 m^2^. The higher serum phosphate level within the normal range was associated with all-cause mortality in the subgroup with eGFR <60 mL/min/1.73 m² (aHR 1.13; 95% CI, 1.02-1.25), indicating a 13% higher risk of death after adjustment for confounders (eg, age, age-squared, sex, race/ethnicity, educational status, smoking status, history of diabetes, history of cardiovascular diseases, history of cancer, body mass index, body mass index-squared, adjusted serum calcium, serum phosphate, eGFR, and the interaction term between serum phosphate level and eGFR) despite null association in the subgroup with eGFR of 60 mL/min/1.73 m^2^ or more (aHR 1.01 [95% CI, 0.92-1.12]; *P* for interaction, .14; Table 5). In the restricted cubic splines for each subgroup, the association of higher serum phosphate levels within the normal range with all-cause mortality was prominent in the subgroup with eGFR < 60 mL/min/1.73 m^2^ (Fig. 3).

**Table 4:** Clinical characteristics among participants included in the subgroup analysis by renal function according to estimated glomerular filtration rate, NHANES III

	Total	Estimated glomerular filtration rate (mL/min/1.73 m^2^		
		≥60	≤30, <60	<30
	n = 15 482	n = 12 327	n = 3018	n = 137
Serum phosphate, mg/dL	3.5 (0.4)	3.5 (0.4)	3.4 (0.4)	3.6 (0.5)
Serum phosphate category				
2.5-2.9 mg/dL	2115 (13.7%)	1642 (13.3%)	462 (15.3%)	11 (8.0%)
3.0-4.0 mg/dL	11 758 (75.9%)	9393 (76.2%)	2266 (75.1%)	99 (72.3%)
4.1-4.5 mg/dL	1609 (10.4%)	1292 (10.5%)	290 (9.6%)	27 (19.7%)
Estimated glomerular filtration rate, mL/min/1.73 m^2^	77.5 (20.8)	84.9 (15.8)	49.6 (7.7)	23.7 (5.8)
Age, years	47.4(20.0)	41.1 (16.5)	71.8 (11.7)	77.2 (10.3)
Sex				
Male	7220 (46.6%)	5926 (48.1%)	1234 (40.9%)	60 (43.8%)
Female	8262 (53.4%)	6401 (51.9%)	1784 (59.1%)	77 (56.2%)
Cardiovascular disease	1274 (8.2%)	574 (4.7%)	651 (21.6%)	49 (35.8%)
Diabetes mellitus	1214 (7.8%)	742 (6.0%)	430 (14.2%)	42 (30.7%)

Data are presented as mean (SD), n (%), or median (interquartile range).

Abbreviation: NHANES III, US Third National Health and Nutrition Examination Survey.

**Table 5:** Association of serum phosphate levels with all-cause mortality in subgroups according to participants’ renal function, NHANES III

	Serum phosphate levels (mg/dL)		
	Lower-normal (≤2.5, <3.5)	High-normal (≤3.5, ≤4.5)	*P* for interaction
**eGFR**			
**≥60 mL/min/1.73 m^2^**			
Number analyzed	5919	6408	0.139
Number of events	1995	1737	
Hazard ratio (95% CI)	Reference	1.01 (0.92-1.12)	
**< 60 mL/min/1.73 m^2^**			
Number analyzed	1600	1555	-
Number of events	1416	1357	
Hazard ratio (95% CI)	Reference	1.13 (1.02-1.25)	

The model included age, age-squared, sex, race/ethnicity, educational status, smoking status, history of diabetes, history of cardiovascular diseases, history of cancer, body mass index, body mass index-squared, adjusted serum calcium, serum phosphate, eGFR, and the interaction term between serum phosphate level and eGFR.

Abbreviations: eGFR, estimated glomerular filtration rate; NHANES III, US Third National Health and Nutrition Examination Survey.

## Discussion

In this cohort study of US nationally representative adults, higher-normal serum phosphate levels within the normal range were associated with increased risk of all-cause mortality among adults with CKD. We found no association among those without CKD. This pattern was also observed in the restricted cubic spline model, suggesting the important role of serum phosphate levels even within the normal range on long-term health among individuals with CKD.

To our knowledge, this is the first study to investigate the association between serum phosphate levels within the normal range and risk of mortality. It has been well established that hyperphosphatemia is associated with increased risk of mortality, especially in patients with CKD [4-11, 24]. A meta-analysis including 47 cohort studies of patients with CKD demonstrated that the risk of death increased 18% for every 1 mg/dL increase in serum phosphate level, whereas there was no significant association between all-cause mortality and serum calcium or PTH [9]. In addition, there is observational evidence that higher serum phosphate levels are associated with increased risk of death, even in individuals with normal kidney function [12-15]. In the present study, the trend was also found in serum phosphate level within the normal range, and the association was even more clearly shown in patients with CKD.

There are several potential mechanisms that would explain the association of higher-normal serum phosphate levels within the normal range with greater risk of mortality. Phosphate is a relatively abundant mineral found in almost all places in the human body. Although it has important physiological roles in maintaining musculoskeletal functions, it can also become pathogenic in extraskeletal system as the term “phosphate toxicity” implies. Phosphate activates inflammatory signaling pathways, particularly through nuclear factor kappa B (NF-kB) [25]. These signals are linked to oxidative stress, which activates a DNA damage response, and can further induce inflammation and promote cell senescence [26-29]. As a result, phosphate induces various pathologies including not only vascular calcification but also increased tumorigenesis, increased cell death, impaired cell signaling, and premature aging [30-33].

Serum phosphate levels are modifiable; however, the factors that determine individual serum phosphate levels are not fully understood. Although definitive evidence is lacking, genetic influences may contribute to individual variations in serum phosphate levels. Daily dietary habits are also likely to play an important role [34]. Similar to the use of phosphate-restricted diets for hyperphosphatemia in patients with kidney dysfunction, serum phosphate levels can be modified by reviewing and adjusting dietary phosphorus intake. Because large amounts of phosphate are contained in processed foods and food additives, public health measures to raise awareness of excessive phosphate consumption are of considerable importance.

Current nephrology guidelines, including those from Kidney Disease Improving Global Outcomes, make weak recommendations for correcting serum phosphate levels toward the normal range in patients with CKD, based on evidence suggesting the association between hyperphosphatemia and increased risk of CVD and mortality [16]. Importantly, the guideline does not address the prognostic significance of serum phosphate levels within the normal range, particularly among individuals with impaired kidney function.

However, no recommendations specify how much to lower serum phosphate levels within the normal range because no studies have explored the relationship between serum phosphate levels within the normal range and various clinical outcomes among patients with CKD [24]. Our study specifically demonstrated that, among participants with reduced renal function, those with higher serum phosphate levels within the normal range had an increased risk of mortality. This finding suggests that even modest elevations in serum phosphate—below the threshold of overt hyperphosphatemia—may have clinical relevance in this population, an aspect not covered by existing guidelines. In this context, our results provide important evidence supporting the consideration of maintaining serum phosphate at the lower end of the normal range for individuals with impaired kidney function.

## Strengths and limitations

The strengths of our study include that we used a nationwide representative sample in the United States, allowing for generalizing the findings to the entire US population. The data from the NHANES III were also linked to the national mortality data with long-term follow-up (ie, median follow-up >25 years), resulting in a sufficient number of outcome events to provide precise estimates.

However, several limitations need to be considered. First, the exposure variable (ie, serum phosphate level) was measured only 1 time point at the survey, and the exact time of blood collection was not available. Although several epidemiologic studies have used a single measurement of serum phosphate evaluate to long-term outcomes [11], we could not account for diurnal variation in serum phosphorus levels, which may have introduced nondifferential measurement error.

Second, the information of the covariates included in our statistical models was measured at the same time point as the exposure variable, serum phosphate levels, in NHANES III, which leaves the temporal ordering of these variables unclear. This may have caused the risk of adjusting for mediators, not confounders, that could have biased the estimates. Third, the risk of confounding bias because of unmeasured confounders cannot be ruled out because of the observational nature of our study. Last, since the information of several covariates collected was self-reported, the risk of information bias due to misclassification remains.

## Conclusions

Among US adults, higher serum phosphate levels within the normal range were associated with increased all-cause mortality only among patients with CKD. Given that randomized controlled trials have not shown the reduction in CVD or mortality from controlling serum phosphate levels using phosphate-binding agents among patients with CKD or hemodialysis [35], future research is needed to determine whether correcting higher-normal serum phosphate levels can reduce long-term adverse health outcome.

## Data Availability

Original data generated and analyzed during this study are included in this published article or in the data repositories listed in References.

## Abbreviations

aHR: adjusted hazard ratio
BMI: body mass index
CKD: chronic kidney disease
CVD: cardiovascular disease
eGFR: estimated glomerular filtration rate
NHANES III: Third National Health and Nutrition Examination Survey
